# Rapid molecular syndromic testing for aetiological diagnosis of gastrointestinal infections and targeted antimicrobial prescription: experience from a reference paediatric hospital in Spain

**DOI:** 10.1007/s10096-021-04266-7

**Published:** 2021-05-08

**Authors:** Marta Castany-Feixas, Silvia Simo, Selene Garcia-Garcia, Mariona Fernandez de Sevilla, Cristian Launes, Marlene Kalkgruber, Amadeu Gene, Carmen Muñoz-Almagro, Pedro Brotons

**Affiliations:** 1 Institut de Recerca Sant Joan de Deu, Barcelona, Spain; 2 CIBER de Epidemiología y Salud Pública CIBERESP, Madrid, Spain; 3 Universitat Internacional de Catalunya, Barcelona, Spain; 4 University Hospital Sant Joan de Deu, P° Sant Joan de Déu, n° 2 08950 Esplugues, Barcelona, Spain; 5 Universitat de Barcelona, Barcelona, Spain

**Keywords:** Diagnosis, Antimicrobial, Gastrointestinal infection, Molecular test, Multiplex PCR, Children

## Abstract

Aetiological diagnosis of gastrointestinal infections is challenging since a wide range of bacteria, parasites and viruses can be causal agents and derived clinical manifestations appear quite similar. Our aim was to evaluate contribution of the novel QIAstat-DxGastrointestinal Panel (GIP) to aetiological diagnosis of gastrointestinal infections and rational antimicrobial prescription in a reference paediatric hospital. Evaluation included comparison of diagnostic yield and agreement of results of QIAstat-Dx GIP and conventional microbiological methods. Parallel testing was performed on stool samples collected prospectively from children admitted to Sant Joan de Deu Barcelona Hospital (Spain) during the period February–March 2019*.* Influence of the panel test use on antimicrobial prescription was assessed using a pre–post study design. Eighty-six (68.8%) out of 125 specimens were positive by QIAstat-Dx GIP versus 44 (35.2%) positive by a composite of conventional methods (*p*<0.001). Global agreement of panel test results with rotavirus-adenovirus antigen detection (92.8%) and a two-step antigen/toxin and PCR-based algorithm for toxigenic *Clostridioides difficile* detection (87.5%) was greater than that with bacterial culture (76.0%) and parasite microscopic identification (64.3%). Panel test results orientated antimicrobial prescription changes in 18 (14.4%) patients, including antimicrobial start in 11 cases initially untreated, targeted antimicrobial prescription in 5 and discontinuation in 2 cases empirically treated. Results showed that QIAstat-Dx GIP significantly expanded aetiological diagnosis of gastrointestinal infections compared to conventional microbiological methods while orientating a more judicious use of antimicrobial drugs in hospitalised children.

**Supplementary Information:**

The online version contains supplementary material available at 10.1007/s10096-021-04266-7.

## Introduction

Diarrhoea remains an important global health problem that was estimated to cause 1.6 million deaths in 2016 [1]. The disease especially affects paediatric populations [2–4] and has been acknowledged as one of the five leading causes of mortality and disability-adjusted life years in children under 5 years of age [5]. Diarrhoea usually occurs in the context of an acute or chronic gastrointestinal infection. Aetiological diagnosis of gastrointestinal infections is challenging since a wide range of bacteria, parasites and viruses can be causal agents [6, 7], and derived clinical manifestations appear quite similar [8].

Traditionally, aetiological diagnosis of gastrointestinal infections has relied on diverse microbiological methods such as stool bacterial culture, parasite microscopic examination, antigen-based detection of specific viruses and nucleic acid amplification for DNA/RNA detection of single highly prevalent pathogens like *Clostridioides difficile* (*C. difficile*) or norovirus. In the last years, new rapid syndromic molecular tests that can simultaneously detect and identify pathogenic gastrointestinal bacteria, viruses and parasites have emerged to overcome limitations of conventional microbiological tests [9–12]. Timely and comprehensive detection of gastrointestinal pathogens is essential to guide targeted antimicrobial treatment, prevent infection transmission and improve clinical outcomes [13].

The QIAstat-Dx Gastrointestinal Panel (Qiagen, Germany), hereafter QIAstat-DxGIP, is a CE-marked molecular panel assay that allows detection of 14 bacteria, 4 parasites and 6 viruses in 1 step in about 1 h. The panel test includes the following targets: *C. difficile* toxin A/B, enteroaggregative *Escherichia coli* (EAEC), enteroinvasive *E. coli* (EIEC)/*Shigella*, enteropathogenic *E. coli* (EPEC), enterotoxigenic *E. coli* (ETEC) lt/st, pathogenic *Campylobacter* spp., *Plesiomonas shigelloides*, *Salmonella* spp., shiga-like toxin producing *E. coli* (STEC) stx1/stx2, shiga-like toxin producing *E. coli* (STEC) O:157:H7, *Vibrio cholera*, *Vibrio parahaemolyticus*, *Vibrio vulnificus*, *Yersinia enterocolitica*, *Cyclospora cayetanensis*, *Cryptosporidium spp*., *Entamoeba histolytica*, *Giardia lamblia*, adenovirus F40/41, astrovirus, norovirus GI, norovirus GII, rotavirus A and sapovirus (I, II, IV and V). This real-time PCR test performs sample preparation and analysis steps automatically within disposable cartridges and yields results in 70 min, including cycle threshold (Ct) values for detected targets. An internal control producing a positive signal validates the results of the test. Conversely, a negative signal from the internal control invalidates all negative results except for those targets positively identified.

The objective of this study was to assess the contribution of QIAstat-Dx GIP to aetiological diagnosis of gastrointestinal infections and antimicrobial stewardship in a reference paediatric hospital.

## Materials and methods

### Study design and setting

A prospective study was conducted to determine usefulness of QIAstat-Dx GIP and conventional microbiological methods for diagnosis of gastrointestinal infections and antimicrobial stewardship in Sant Joan de Deu Barcelona Hospital (Spain) during February–May 2019. The setting is a 318-bedsize reference university paediatric hospital that attends a population of approximately 300,000 children. Inclusion criteria were as follows: (1) use of fresh loose stool samples collected from patients ≤18 years of age hospitalised in the study site with suspicion of gastrointestinal infection and diarrhoea symptomatology; (2) stool testing by QIAstat-Dx GIP, bacterial culture and rotavirus-adenovirus antigen detection, as well as microscopic examination for identification of parasites and performance of a 2-step antigen/toxin and PCR-based algorithm for toxigenic *C. difficile* detection in specific patients; and (3) selection of the first stool sample produced by each patient. Impact of the panel test utilisation on antimicrobial prescribing decisions was evaluated using a pre–post study design.

Outcomes sought were diagnostic yield of QIAstat-Dx GIP and conventional pathogen-targeted tests, agreement between tests and effect of the new syndromic test use on antimicrobial prescription changes.

### Diagnostic methods and interpretation of results

Stool specimens were collected and routinely processed on demand by bacterial culture to identify *E. coli*, *Campylobacter* spp., *Plesiomonas shigelloides*, *Salmonella* spp., *Vibrio cholera*, *Vibrio parahaemolyticus*, *Vibrio vulnificus* and *Yersinia enterocolitica*. Culturing was performed according to standard operational procedures of the study setting. Identification of rotavirus and adenovirus was carried out using the antigen SD Rota/Adeno Rapid test following the manufacturer’s instructions. Parasites including *Cryptosporidium* spp., *Cyclospora cayetanensis*, *Entamoeba histolytica* and *Giardia lamblia* were identified by microscopy observation of their morphological characteristics. Toxigenic *C. difficile* detection combined the use of the C. Diff QuikChek Complete test (Abbott, USA) for *C. difficile* antigen and toxin detection and, if it was the case, the PCR-based GeneXpert® CD assay (Cepheid, USA) for confirmation of *C. difficile* antigen-positive and toxin-negative results, according to instructions of manufacturers. Microscopy observations for parasites and sequential tests for toxigenic *C. difficile* infection were only requested for a determinate number of samples at the clinician’s discretion. All samples were also tested by QIAstat-Dx GIP in parallel.

A multi-disciplinary board including a paediatrician, a paediatric infectious diseases specialist and a microbiologist jointly interpreted results of tests and reassessed diagnostic classifications and adequacy of baseline antimicrobial prescriptions.

### Statistical analysis

Percent agreement between results by QIAstat-Dx GIP and conventional methods and kappa coefficients were calculated as described elsewhere [14]. Differences in proportions of positivity rates between tests and extent of antimicrobial prescription changes due to QIAstat-Dx GIP results were determined by the Chi-square or the exact test. Statistical significance was set at a *p*-value of <0.05 and confidence intervals (CI) at 95% level. All statistical analyses were performed using Stata v.15.1 software (Stata Corp.).

## Results

### Sample testing methods and patient characteristics

A total of 146 stool specimens were collected during the study period. Twenty-one (14.4%) of them were discarded, including 15 samples not tested by culture and/or rotavirus-adenovirus antigen detection, 5 samples that yielded invalid results by QIAstat-Dx GIP (negative for the internal control and for any target) and 1 sample not tested by the panel test. One hundred and twenty-five specimens from 125 patients were finally included in the study and underwent QIAstat-Dx GIP as well as conventional stool culture and rotavirus-adenovirus antigen testing. Additionally, 24 (19.2%) and 14 (11.2%) samples were tested for antigen/toxin- and PCR-based toxigenic *C. difficile* detection and parasite identification, respectively.

Seventy-three (58.4%) patients were male. Median age of patients was 20.4 months (IQR, 8.0–65.9 months). A majority of participants were recruited in the general paediatric ward (n=81, 64.8%), followed by those staying in haematology (n=12, 9.6%) and oncology (n=9, 7.2%) wards.

### Laboratory diagnostic results

Eighty-six (68.8%) out of 125 specimens were found positive by QIAstat-Dx GIP. A single pathogen was detected in 50 (58.1%) specimens whereas co-detection of 2, 3 and 4 pathogens was observed in 26 (30.2%), 8 (9.3%) and 2 (2.3%) samples, respectively. A total of 134 pathogens were identified in the 86 positive samples, rotavirus being the most prevalent species (n=40), followed by toxigenic *C. difficile* (n=17) and norovirus GII (n=14). QIAstat-Dx GIP identified co-infections in 36 (28.8%) specimens. Fourteen samples positive for toxigenic *C. difficile* corresponded to children aged less than 2 years*.* Mean time to result was 10 h (IQR, 4–24 h).

**Fig. 1:**
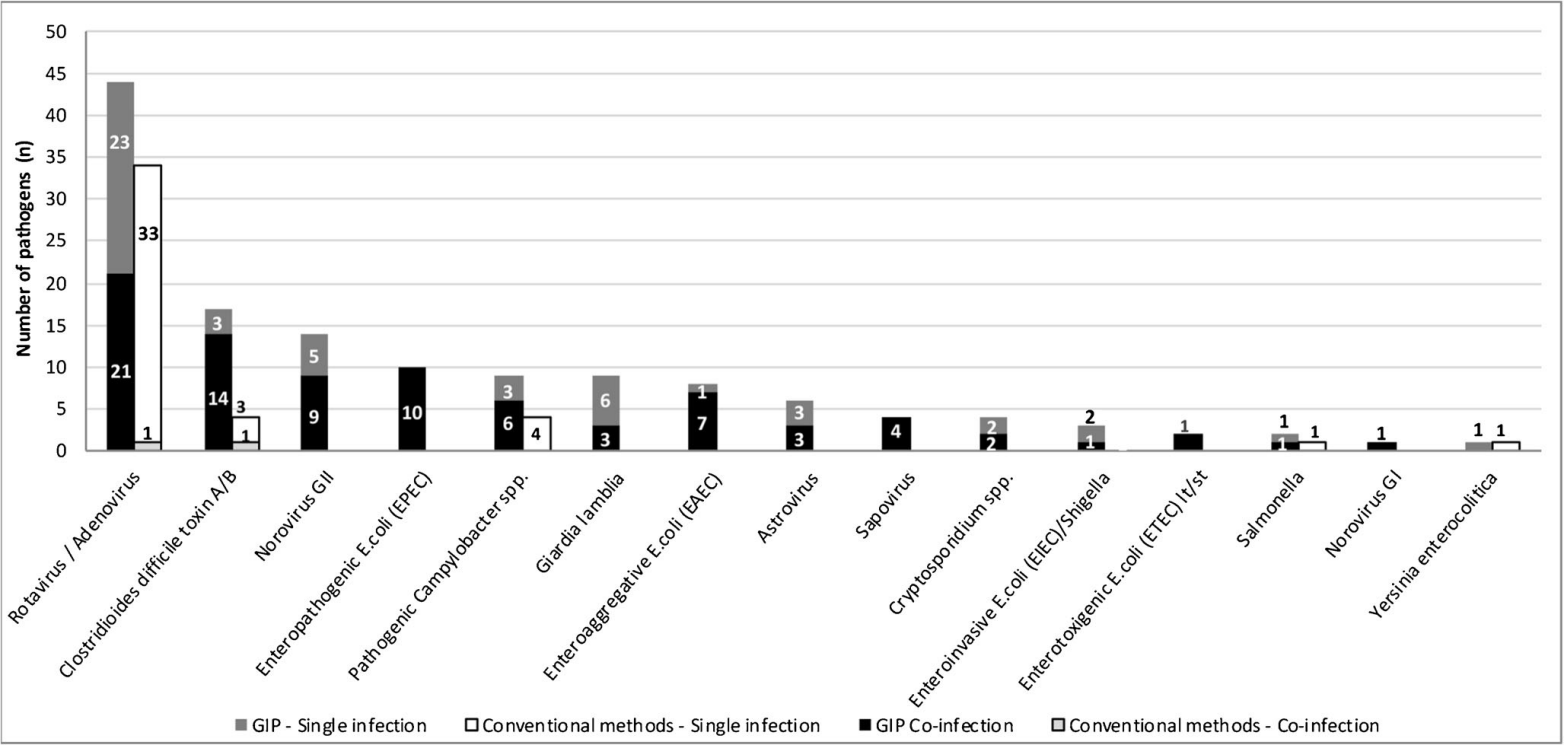
Pathogen distribution by QIAstat GIP and conventional microbiological methods

Forty-four (35.2%) samples yielded positive results and 45 pathogens were identified by conventional microbiological methods. Rotavirus-adenovirus was found in 34 (27.2%) out of the 125 samples and toxigenic *C. difficile* in 4 (16.7%) out of 24 samples, either directly by C. Diff QuikChek Complete antigen and toxin detection test (n=1) or after GeneXpert CD confirmation (n=3). Colonies of *Campylobacter* (n=4, 3.2%), *Salmonella* (n=1, 0.8%) and *Yersinia* (n=1, 0.8%) grew by stool culture. Only one bacterial-viral co-infection by toxigenic *C. difficile* and rotavirus-adenovirus was identified by the set of traditional diagnostic methods. Positivity rates for pathogen groups and at pathogen-level are detailed in Table 1. Overall distribution of pathogens by QIAstat-Dx GIP and conventional microbiological methods is depicted in Fig. 1. Supplementary Table 1 describes pathogen combinations identified by QIAstat-Dx GIP in co-infected samples.

**Table 1:** Positivity rates by QIAstat-Dx GIP and conventional microbiological methods

Pathogen^a^	QIAstat-Dx GIP		Conventional method		
	No. of samples	No. of positives (%)	No. of samples	No. of positives (%)	*p* value
Groups of pathogens shared between compared tests					
Any identifiable pathogen (QIAstat vs. composite reference)	125	86 (68.8)	125	44 (35.2)	<0.001
Rotavirus-adenovirus (QIAstat vs. antigen and toxin detection)	125	43 (34.4)	125	34 (27.2)	0.22
Any pathogenic bacteria (QIAstat vs. stool culture)	125	38 (30.4)	125	6 (4.8)	<0.001
Any parasite (QIAstat vs. microscopic examination)	125	13 (10.4)	14	1 (7.1)	0.70
Individual pathogens shared between compared tests					
*C. difficile* toxin A/B (QIAstat vs. 2-step detection algorithm)	125	17 (13.6)	24	4 (16.7)	0.69
Pathogenic *Campylobacter* spp.	125	9 (7.2)	125	4 (3.2)	0.15
*Giardia lamblia*	125	9 (7.2)	14	0 (0.0)	0.30
*Cryptosporidium* spp.	125	4 (3.2)	14	0 (0.0)	0.50
*Salmonella*	125	2 (1.6)	125	1 (0.8)	0.56
*Yersinia enterocolitica*	125	1 (0.8)	125	1 (0.8)	1.00
Pathogens not shared between compared tests					
EPEC	125	10 (8.0)	-	-	NA
EAEC	125	8 (6.4)	-	-	NA
EIEC/Shigella	125	3 (2.4)	-	-	NA
ETEC lt/st	125	2 (1.6)	-	-	NA
STEC stx1/stx2	125	0 (0.0)	-	-	NA
STEC O157:H7	125	0 (0.0)	-	-	NA
Norovirus GII	125	14 (11.2)	-	-	NA
Astrovirus	125	6 (4.8)	-	-	NA
Sapovirus (I, II, IV, V)	125	4 (3.2)	-	-	NA
Norovirus GI	125	1 (0.8)	-	-	NA

Values expressed as No. (%)

*Abbreviations*: *C. difficile*, *Clostridioides difficile*; *EPEC*, enteropathogenic *Escherichia coli*; *EAEC*, enteroaggregative *E. coli*; *ETEC*, enterotoxigenic *E. coli*; *EIEC*, enteroinvasive *E. coli*; *STEC*, Shiga-like toxin producing *E. coli*; *NA*, not applicable

^a^*Plesiomonas shigelloides*, *Vibrio cholera*, *Vibrio parahaemolyticus*, *Vibrio vulnificus*, *Cyclospora cayetanensis* and *Entamoeba histolytica* were not identified by any test

Overall agreement of QIAstat-Dx GIP results with those of individual conventional techniques ranged from high for rotavirus-adenovirus (92.8%) and toxigenic *C. difficile* (87.5%) identification, to fair for bacteria identification by stool culture (76.0%), to poor for parasite identification by microscopy (64.3%). Overall, positive and negative percent agreement values and kappa coefficients are detailed in Table 2. There were 13 discrepant results in samples tested by both QIAstat-DX GIP and conventional methods for detection of shared targets, including 5 positives for *Campylobacter* spp. by QIAstat-DX GIP and negative by stool culture, 4 positives for *Giardia lamblia* by QIAstat-DX GIP and negative by microscopy, two positives for toxigenic *C. difficile* by QIAstat-DX GIP and negative by the 2-step algorithm, one negative for toxigenic *C. difficile* by QIAstat-DX GIP and positive by the 2-step algorithm, and one positive for *Salmonella* spp. by QIAstat-DX GIP and negative by stool culture.

**Table 2:** Agreement rates between QIAstat-Dx GIP and conventional microbiological methods

Diagnostic test	Target	No. of samples	Global agreement	95% CI	Positive agreement	95% CI	Negative agreement	95% CI	Kappa coefficient
QIAstat vs. antigen and toxin detection	Rotavirus-adenovirus	125	92.8	86.9–96.2	88.3	79.3–93.7	94.8	90.4–97.2	0.83
QIAstat vs. stool culture	Culturable bacteria	125	76.0	67.8–82.6	34.8	22.7–49.2	85.3	79.8–89.5	0.27
QIAstat vs. 2-step detection algorithm	*C. difficile* toxin A/B	24	87.5	69.0–95.7	66.7	35.4–87.9	92.3	79.7–97.3	0.59
QIAstat vs. microscopic examination	Parasites	14	64.3	38.8–83.7	0.0	0.0–43.4	78.3	58.1–90.3	−0.13

Values expressed as percentages, unless otherwise stated

*Abbreviations*: *CI*, confidence interval

### Contribution of QIAstat-Dx GIP to targeted antimicrobial prescription

More comprehensive aetiological diagnosis of gastrointestinal infections by the panel test led to antimicrobial treatment changes for 18 (14.4%) out of the 125 patients, representing a significant effect (*p*<0.001) in comparison to baseline prescriptions. Eleven out of 83 patients that had not initially been treated with antimicrobials started receiving them upon identification of bacterial (n=5), parasitic (n=5) and bacterial-parasitic co-infections (n=1), including two patients younger than 2 years of age that were found positive for toxigenic *C. difficile* by QIAstat-Dx GIP. Among the remainder 42 patients that received antimicrobials empirically on admission, 5 were prescribed a more targeted therapy following identification of bacterial (n=4) and parasitic (n=1) infections*,* and 2 had antimicrobials discontinued once a viral aetiology had been confirmed. Seven (38.9%) out of the 18 antimicrobial prescription changes affected onco-haematology inpatients, a subgroup with a significantly lower participation weight (16.8%, *p*=0.03) in relation to the total study population. Details of pathogens identified before and after panel testing and subsequent antimicrobial treatment changes are explained in Table 3.

**Table 3:** Antimicrobial prescription changes due to QIAstat-Dx GIP use

Hospital ward	Patient age (years)	Targets identified before QIAstat testing	Targets identified after QIAstat testing	Pre-test antimicrobial prescription	Post-test antimicrobial prescription	Type of antimicrobial prescription change
Paediatrics	0.9	None	Toxigenic *C. difficile*, EAEC	None	Metronidazol	Antimicrobial start
Paediatrics	6.8	None	*Salmonella*	None	Ceftriaxone	Antimicrobial start
Paediatrics	1.3	None	*G. lamblia*	None	Metronidazol	Antimicrobial start
Nephrology	18.4	None	*G. lamblia*, norovirus GI	None	Metronidazol	Antimicrobial start
Paediatrics	0.2	None	*Campylobacter*	None	Azitromicine	Antimicrobial start
Paediatrics	1.3	None	Toxigenic *C. difficile + Cryptosporidium*	None	Nitazoxanide	Antimicrobial start
Haematology	6.2	None	*G. lamblia*	None	Metronidazol	Antimicrobial start
Intensive Care Unit	0.1	None	Cryptosporidium	None	Paramomicine	Antimicrobial start
Paediatrics	2.0	None	EPEC *+ Campylobacter*	None	Azitromicine	Antimicrobial start
Gastroenterology	2.6	None	*G. lamblia*	None	Metronidazol	Antimicrobial start
Gastroenterology	10.8	None	Toxigenic *C. difficile*	None	Metronidazol	Antimicrobial start
Haematology	18.3	None	EPEC, ETEC, *Salmonella*	Ceftriaxone	Amoxicilline	Targeted antimicrobial
Paediatrics	2.7	None	*G. lamblia*	Ceftriaxone	Ceftriaxone + metronidazol	Targeted antimicrobial
Oncology	6.7	*Campylobacter*	Toxigenic *C. difficile + Campylobacter*	Ceftriaxone	Azitromicine + metronidazol	Targeted antimicrobial
Haematology	9.3	None	Toxigenic *C. difficile*	Amoxicillin-clavulanic	Amoxicillin-clavulanic + metronidazol	Targeted antimicrobial
Oncology	2.0	None	EPEC *+ Campylobacter*	Meropenem	Azitromicine	Targeted antimicrobial
Oncology	2.3	None	Astrovirus	Piperacillin/tazobactam	0	Antimicrobial discontinuation
Haematology	14.0	None	Norovirus GII	Meropenem	0	Antimicrobial discontinuation

*Abbreviations*: *C. difficile*, *Clostridioides difficile*; *G. lamblia*, *Giardia lamblia*; *EAEC*, enteroaggregative *Escherichia coli*; *EPEC*, enteropathogenic *E. coli*; *ETEC*, enterotoxigenic *E. coli*

## Discussion

As far as we know, our study is the first to report a significant influence of the use of a syndromic molecular test, the QIAstat-Dx GIP, on antimicrobial prescription practices for children hospitalised with suspected gastrointestinal infections. Interestingly, Beal et al. documented a trend for a lower intensity of antibiotic utilisation that did not reach statistical significance when comparing two groups of hospitalised children tested by another multiplex molecular assay, the FilmArray Gastrointestinal Panel (FA GIP), and stool culture [15]. We speculate that the comparatively higher effect of syndromic molecular testing on antimicrobial prescription practices observed in our study could partly be due to the noticeable number of children with oncology-haematology conditions, since this subgroup was subject to approximately 1.5 times more post-test antimicrobial prescription changes than the rest of participants.

Among inpatients of all ages, Cybulski et al. described that subjects diagnosed by FA GIP were significantly more prone to receive targeted than empirical therapy compared to those diagnosed by culture [16], whereas Axelrad et al. observed a significant reduction in antibiotic prescription within 14 days since stool testing after implementation of FA GIP, in comparison with conventional stool culture and antigen detection of adenovirus, rotavirus and parasites [11]. In contrast, Freeman et al. in their systematic review of syndromic molecular testing for GI did not identify robust evidence of antimicrobial optimisation and other clinical impacts following the introduction of panel tests [17].

The new syndromic test was able to unveil aetiology of gastrointestinal infections for the majority of study patients and had a nearly two-fold higher detection rate than targeted microbiological assays. Recent studies comparing QIAstat-Dx GIP with FA GIP and laboratory-developed RT-PCR assays reported similar diagnostic performance by the compared tests for common targets [18, 19]. Other comparative studies between multiplex molecular tests have also described similar capabilities to identify gastrointestinal pathogens [20, 21]. We hypothesise that the positive results yielded by QIAstat GIP in 9 samples (5 negative for *Campylobacter* spp. by stool culture and 4 negative for *Giardia lamblia* by microscopy observation) may probably be true positives, since real-time PCR tests like QIAstat GIP are widely acknowledged to have higher accuracy than the conventional methods that were used as comparators. A balance of discrepancies was observed between results by QIAstat GIP and the 2-step antigen- and PCR-based method for detection of toxigenic *C. difficile* in 3 samples (two pairs of positive results by the former method and negative by the latter, one pair of negative results by the former method and positive by the latter). This balance could indicate that both methods have equivalent diagnostic accuracy, as reflected by the high overall agreement of their results (92.8%). Generally, syndromic molecular testing appears to correctly identify gastrointestinal pathogens in concordance with results of conventional microbiological methods while additionally finding a larger number of pathogenic species and co-infections that would otherwise have been missed [11, 16, 17].

Studies on asymptomatic carriers have raised considerable uncertainty about whether or not additional gastrointestinal infection-positive results delivered by panel tests may be clinically important. Presence of *C. difficile* or its toxin in children under 2 years of age may be confounding since up to 70% of healthy newborns are colonized asymptomatically, a rate that gradually falls in parallel to the establishment of the intestinal microbiota by about that age [22]. Pathogenicity of EPEC and EAEC is also open to debate [23], given that these pathotypes have commonly been found in immunosuppressed cancer patients [24]. So is the identification of gastrointestinal viruses, as noticeable carriage rates of gastrointestinal viruses have been observed in asymptomatic children attending daycare centres [25]. Similarly, identification of *Cryptosporidium* and *Giardia* parasites in paediatric stool samples needs careful interpretation in light of non-negligible asymptomatic carriage rates reported among preschool children in a developed country [26] and the lack of a significant association between *Giardia* and diarrhoea documented in children up to 2 years of age in resource-limited settings [27]. In this regard, QIAstat-Dx GIP reporting of pathogen cycle threshold (Ct) values appears as an interesting feature that might contribute to discern the pathogenic or commensal role of specific gastrointestinal microorganisms. In addition, given the risk of antibiotic overuse or misuse in symptomatic patients, a prudent and cost-effective diagnostic strategy could be to prioritise patients with serious comorbidities or a deteriorating clinical course over milder patients for rapid testing by QIAstat-Dx GIP. Moreover, on-demand test use should be based on efficient pre- and post-analytical operational procedures to ensure that turnaround time for results keeps close to QIAstat-Dx GIP 70-min process time.

Interestingly, GI prevalence (68.8%) and co-infection (28.8%) rates determined by QIAstat-Dx GIP were remarkably higher than those reported in previous syndromic testing studies on paediatric study populations. In particular, Stockmann et al. reported a GI prevalence of 52% and a co-infection rate of 15% among children inpatients and outpatients with median age of 5 years [28], whereas Beckmann et al. determined the same prevalence rate for a subgroup of inpatients and outpatients predominantly aged 1–5 years [29]. We hypothesise that patient demographic and clinical characteristics as well as local epidemiology of GI and seasonal effects may explain these differences, since infants <2 years were significantly more prone to co-infection in our study than older children.

This study has some limitations for generalisation of results. First, it was a single-centre observational study with a relatively small sample size. Second, our study population included 14 infants under 2 years of age positive for toxigenic *C. difficile* by QIAstat-Dx GIP who might have been carriers and not cases. However, a potential bias in the real contribution of QIAstat-Dx GIP to targeted antimicrobial use in those infants was unlikely, since administration of antibiotics was only started in two of them initially untreated as a consequence  of a positive result for *C.difficile* by the panel test. Third, we could only assess agreement of QIAstat-Dx GIP with conventional microbiological assays for specific targets only covered by the panel test. Four, re-testing of samples yielding discrepant results was not performed.

In conclusion, QIAstat-DxGIP use significantly improved aetiological diagnosis of gastrointestinal infections in hospitalised children in comparison with conventional microbiological methods. The contribution of this syndromic test to orientate specific antimicrobial prescription was also remarkable, particularly in onco-haematology paediatric inpatients. Further studies from multi-centre and experimental approaches are needed to confirm clinical impact of the new test.

## Supplementary Information

Supplementary Table 1 **Pathogen combinations identified by QIAstat-Dx GIP in co-infected samples.** Values expressed as No. (%). Abbreviations: *C. difficile*, *Clostridioides difficile*; EPEC, enteropathogeni c*Escherichia coli*; EAEC, enteroaggregative *E. coli*; ETEC, enterotoxigenic *E. coli*; *G. lamblia*, *Giardia lamblia (DOCX 13 kb)*

## Data Availability

The dataset generated during the current study is available from the corresponding author on reasonable request.
