# Supplementary material for: Rapid molecular syndromic testing for aetiological diagnosis of gastrointestinal infections and targeted antimicrobial prescription: experience from a reference paediatric hospital in Spain
Source: Eur J Clin Microbiol Infect Dis. 2021 May 8;40(10):2153–60. doi: 10.1007/s10096-021-04266-7 (PMC8449766; doi:10.1007/s10096-021-04266-7)
Supplement: Supplementary file 1 — Pathogen combinations identified by QIAstat-Dx GIP in co-infected samples. Values expressed as No. (%). Abbreviations: C. difficile, Clostridioides difficile; EPEC, enteropathogeni cEscherichia coli; EAEC, enteroaggregative E. coli; ETEC, enterotoxigenic E. coli; G. lamblia, Giardia lamblia (DOCX 13 kb) [file 10096_2021_4266_MOESM1_ESM.docx]

| **Supplementary Table 1 Pathogen combinations identified by QIAstat-Dx GIP in co-infected samples** | |
| --- | --- |
| Pathogens | No. (%) |
|  |  |
| Co-infection by 4 pathogens | **2 (1.6)** |
| Toxigenic *C. difficile*, EPEC, rotavirus, astrovirus | 1 (0.8) |
| Toxigenic *C. difficile*, *Cryptosporidium spp*, adenovirus, norovirus | 1 (0.8) |
| Coinfection by 3 pathogens | **8 (6.4)** |
| EAEC, Pathogenic *Campylobacter*, sapovirus | 1 (0.8) |
| Rotavirus, astrovirus, sapovirus | 1 (0.8) |
| *C. difficile*, EPEC, rotavirus | 1 (0.8) |
| *C. difficile*, EAEC, rotavirus | 1 (0.8) |
| C. difficile, EPEC, norovirus | 1 (0.8) |
| EAEC, EPEC, rotavirus | 1 (0.8) |
| *C. difficle*, pathogenic *Campylobacter*, rotavirus | 1 (0.8) |
| EPEC, ETEC, Salmonella | 1 (0.8) |
| Coinfection by 2 pathogens | **26 (20.8)** |
| *C. difficile*, norovirus | 4 (3.2) |
| EAEC, rotavirus | 3 (2.4) |
| Rotavirus, sapovirus | 2 (1.6) |
| *G. lamblia*, rotavirus | 2 (1.6) |
| EPEC, pathogenic *Campylobacter* | 2 (1.6) |
| *C. difficile,* EAEC | 1 (0.8) |
| ETEC, rotavirus | 1 (0.8) |
| *G. lamblia,* norovirus | 1 (0.8) |
| EPEC, rotavirus | 1 (0.8) |
| *C. difficle,* pathogenic *Campylobacter* | 1 (0.8) |
| Rotavirus, astrovirus | 1 (0.8) |
| Rotavirus, norovirus | 1 (0.8) |
| Adenovirus, rotavirus | 1 (0.8) |
| *C. difficile, rotavirus* | 1 (0.8) |
| *C. difficile,* Cryptosporidium | 1 (0.8) |
| Pathogenic *Campylobacter,* norovirus | 1 (0.8) |
| EIEC, EPEC | 1 (0.8) |
| EPEC, norovirus | 1 (0.8) |
| Total co-infections | **36 (28.8)** |
